# Supplementary material for: No compromise in efficiency from the co-application of a marine and a terrestrial CDR method
Source: Nat Commun. 2025 May 21;16:4709. doi: 10.1038/s41467-025-59982-x (PMC12092840; doi:10.1038/s41467-025-59982-x)
Supplement: Supplementary file 1 — Supplementary Information [file 41467_2025_59982_MOESM1_ESM.pdf]

Supplementary Information for: No compromise in efficiency from the co-application of a marine and a terrestrial CDR method

Yiannis Moustakis[1*], Hao-Wei Wey[2], Tobias Nützel[1], Andreas Oschlies[2,3], Julia Pongratz[1,4]

[1] Ludwig-Maximilians-Universität in Munich, Munich, Germany

[2] GEOMAR Helmholtz Centre for Ocean Research Kiel, Kiel, Germany

[3] Kiel University, Kiel, Germany

[4] Max Planck Institute for Meteorology, Hamburg, Germany

[*]corresponding author, email: yiannis.moustakis@geographie.uni-muenchen.de

Supplementary Tables

Table S 1: ***Removal efficiency estimates in the literature****: The table shows changes in atmosphere (C_{atmo}), land (C_{land}), and ocean (C_{ocean}) carbon ($GtCO_2$) for the different experiments of past studies studies that have employed Earth System Models (ESMs) and Earth System Models of Intermediate Complexity (EMICs) to investigate the effect of large-scale Carbon Dioxide Removal (CDR) on the Earth system. All values have been obtained from the corresponding studies, and the removal efficiency (%) is then calculated. For the case of Afforestation/Reforestation (AR), removal efficiency is estimated as C_{atmo}/C_{land}, whereas for Ocean Alkalinity Enhancement (OAE), removal efficiency is estimated as C_{atmo}/C_{ocean} (see Methods). For each study, the model used, the experiment name and the emission pathway are also shown.*

	Model	Method	Experiment	Emissions	C_{atmo} removal ($GtCO_2$)	C_{land} change ($GtCO_2$)	C_{ocean} change ($GtCO_2$)	Removal efficiency (%)
Keller et al.[1]	Uvic EMIC	OAE	OA	RCP8.5	609	-55	664	**92%**
		AR	AF		385	481	-95	**80%**
Feng et al.[2]	Uvic EMIC	OAE	OlivInf_con	RCP8.5	3,420	-588	4,008	**85%**
			Oliv10_con		3,186	-503	3,688	**86%**
			Oliv100_con		1,470	-148	1,618	**91%**
			Oliv1000_con		196	-17	213	**92%**
			OlivInf_con_t		1,979	-315	2,294	**86%**
			Oliv10_con_t		2,033	-308	2,341	**87%**
			Oliv100_con_t		1,166	-115	1,281	**91%**
			Oliv1000_con_t		156	-13	169	**92%**
			OlivInf_Omega3.4		964	-93	1,057	**91%**
			Oliv10_Omega3.4		971	-93	1,064	**91%**
			Oliv100_Omega3.4		781	-70	851	**92%**
			Oliv1000_Omega3.4		183	-15	198	**92%**
			OlivInf_Omega9		2,689	-401	3,090	**87%**
			Oliv10_Omega9		2,898	-453	3,351	**86%**
			Oliv100_Omega9		1,450	-143	1,593	**91%**
			Oliv1000_Omega9		196	-17	213	**92%**
			Average					**90%**
Lenton et al.[3]	Uvic EMIC	OAE	AOA_G	RCP8.5	655	-21	677	**97%**
			AOA_SP		673	-18	690	**97%**
			AOA_ST		663	-16	679	**98%**
			AOA_T		640	-10	650	**98%**

			Average RCP8.5					**98%**
			AOA_G	RCP2.6	444	-81	525	**85%**
			AOA_SP		444	-88	533	**83%**
			AOA_ST		447	-78	525	**85%**
			AOA_T		426	-85	511	**83%**
			Average RCP2.6					**84%**
Sonntag et al.[4,5]	MPI-ESM (CMIP5)	OAE	CE-ocean	RCP8.5	3321	-132	3453	**96%**
	MPI-ESM (CMIP5)	AR	CE-land		661	793	-132	**83%**
Koch et al.[6]	HadGEM2-ES	AR	esmrcp26restor	RCP2.6	66.06	121.11	-55.05	**55%**
Loughran et al.[7]	ACCESS-ESM1.5	AR	esm-ssp585-ssp126Lu	SSP5-8.5	86.979	91.75	-4.771	**95%**
Palmiéri & Yool[8]	UKESM1	OAE	EXP1	SSP5-8.5	66.83	-19.89	86.72	**77%**
			EXP2		25.45	-18.94	44.39	**57%**
			EXP3		172.66	4.66	167.99	**103%**
			EXP4		81.09	-13.49	94.58	**86%**
			EXP5		59.85	-24.64	84.49	**71%**
			Average					**79%**
Moustakis et al.[9]	MPI-ESM (CMIP6)	AR	AR	SSP5-3.4os	281	382	-101	**74%**
Jeltsch-Thömmes et al.[10]	Bern3D-LPX	OAE	SSP5-3.4 + OAE	SSP5-3.4os	Removal Efficiency (RE) is estimated as follows: $RE(\%) = \frac{\varepsilon^{e}_{\delta atm}}{\varepsilon^{e}_{\delta ocean}} = \frac{\frac{\Delta C_{atmo}}{Pmol\ added}}{\frac{\Delta C_{ocean}}{Pmol\ added}} = \frac{\Delta C_{atmo}}{\Delta C_{ocean}}$ Values are obtained from Table 1 of Jeltsch-Thömmes et al. (2024)			**87%**
	Uvic EMIC		SSP5-3.4 + OAE	SSP5-3.4os				**73%**
Wey et al.[11]	ACCESS-ESM1-5	AR	esm-ssp585-ssp126Lu	SSP5-8.5	Removal Efficiency (RE) is estimated as follows: $RE(\%) = \frac{\Delta C_{atmo}}{\Delta C_{land}} = \frac{\Delta C_{land} + \Delta C_{ocean}}{\Delta C_{land}} = 100\% + \frac{\Delta C_{ocean}}{\Delta C_{land}} = 100\% + \lambda_{A/R}$ Values for the "carbon-cycle feedback ratio" $\lambda_{A/R}$ are obtained from Table S1 of Wey et al. (2025)			**88%**
	CESM2							**86%**
	CanESM5							**75%**
	FOCI							**83%**
	MIROC-ES2L							**87%**
	MPI-ESM							**85%**
	UKESM							**86%**
	FOCI	OAE	esm-ssp585-ocn-alk	SSP5-8.5				**84%**
	MPI-ESM							**91%**

	NorESM2-LM				$RE(\%) = \frac{\Delta C_{atmo}}{\Delta C_{ocean}} = \frac{\Delta C_{land} + \Delta C_{ocean}}{\Delta C_{ocean}} = 100\% + \frac{\Delta C_{land}}{\Delta C_{ocean}} = 100\% + \lambda_{OAE}$	85%
	UKESM				Values for the “carbon-cycle feedback ratio” λ_{OAE} are obtained from Table S1 of Wey et al. (2025)	88%

Supplementary Figures

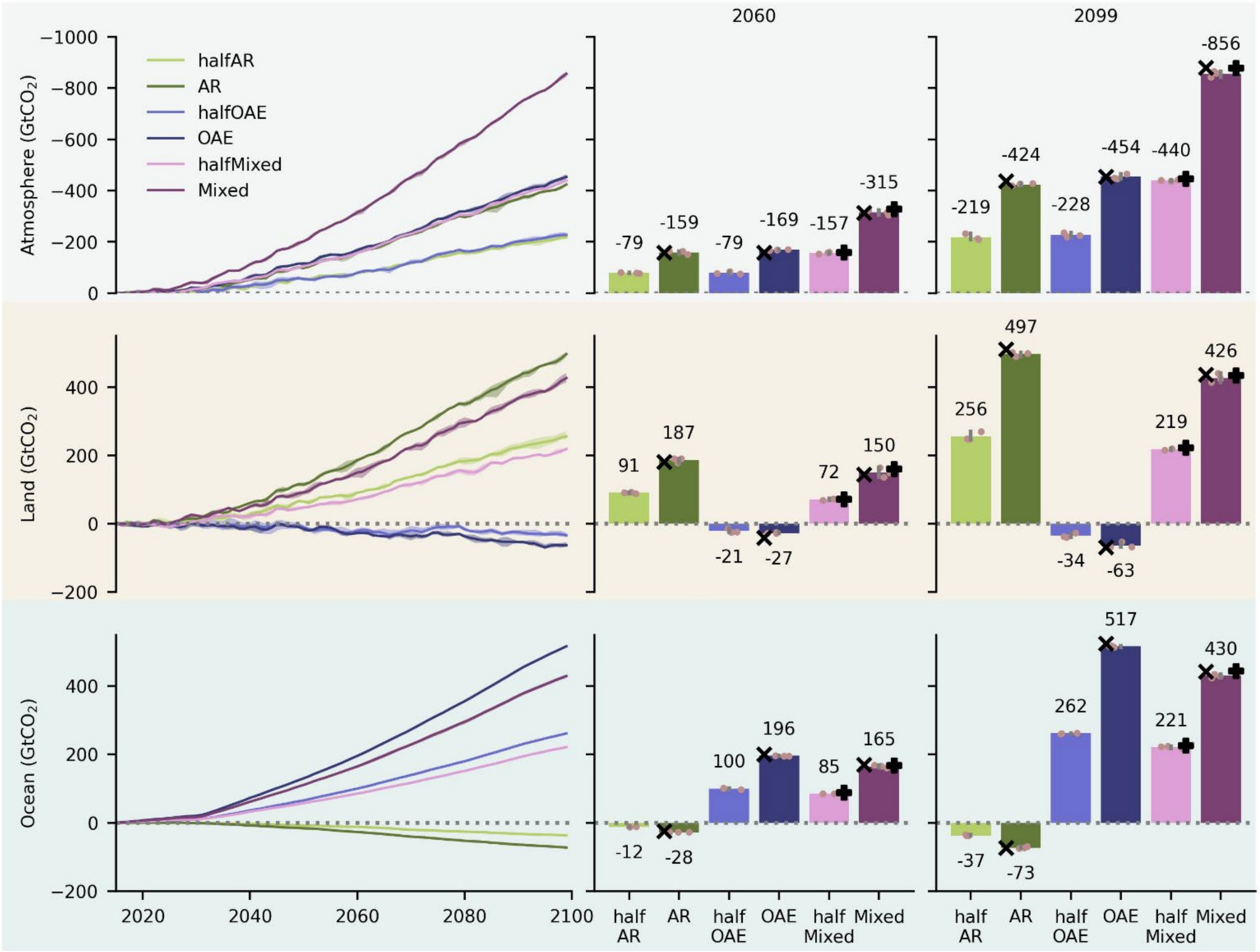

*Figure S 1: **Carbon sequestration in MPI-ESM**: The left column panels show the timeseries of MPI-ESM average change in (top) atmosphere, (middle) land, and (bottom) ocean carbon for all the different Carbon Dioxide Removal (CDR) scenarios compared to REF ($GtCO_2$). The shading around the mean shows the minimum-maximum range across all ensemble members. To aid interpretation, in the middle and right column panels the barplots show snapshots for 2060 and 2099. The bar height corresponds to the average, and the gray vertical lines show the minimum-maximum range across all ensemble members, while the individual data points are also plotted. The multiplication and plus signs indicate expectations from scaling up CDR application and combining methods respectively, based on linearity assumptions (see Table 1). To aid visualization, the vertical axis is flipped in the top panel.*

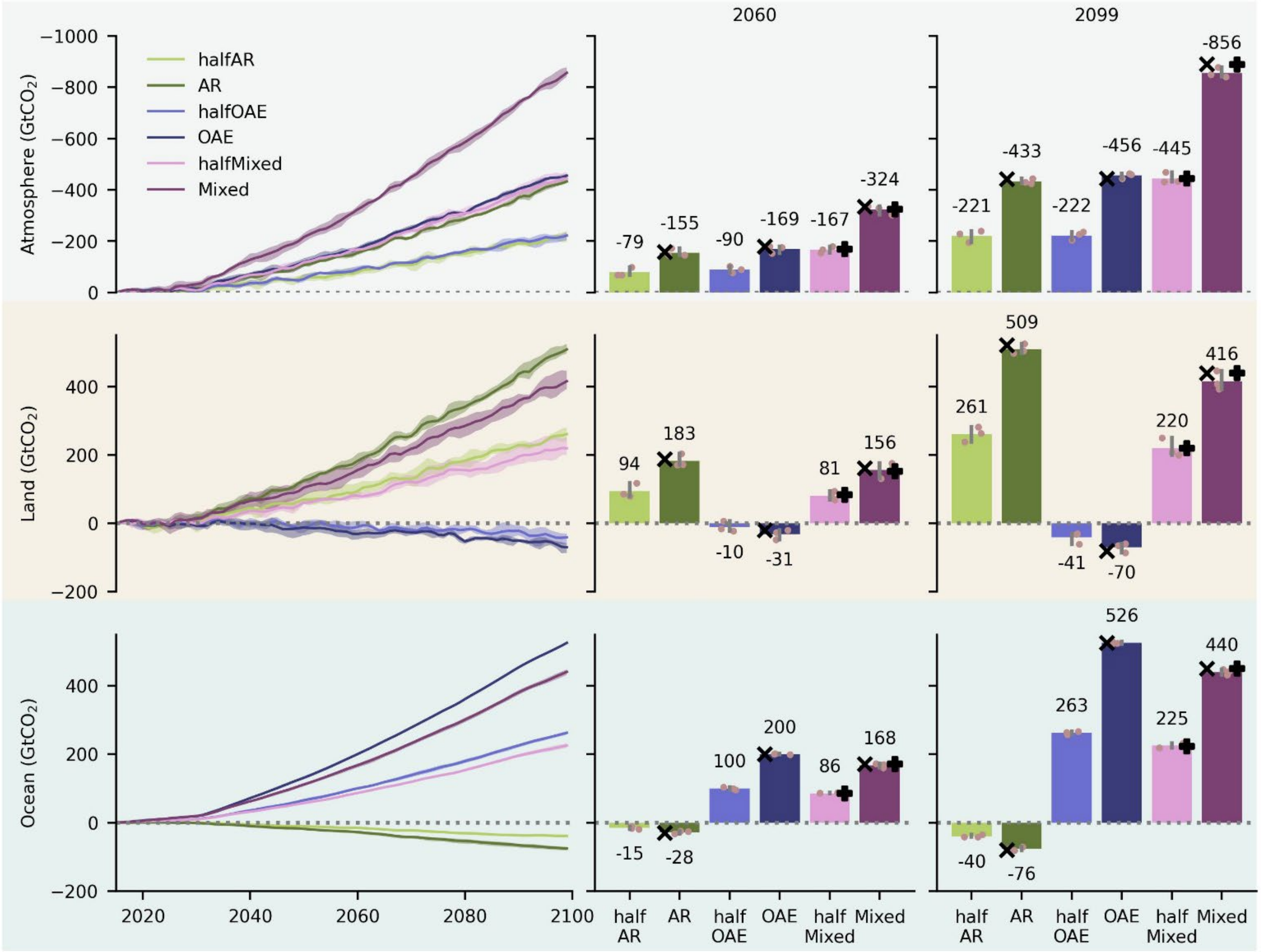

Figure S 2: ***Carbon sequestration in FOCI****: The left column panels show the timeseries of FOCI average change in (top) atmosphere, (middle) land, and (bottom) ocean carbon for all the different Carbon Dioxide Removal (CDR) scenarios compared to REF (GtCO$_2$). The shading around the mean shows the minimum-maximum range across all ensemble members. To aid interpretation, in the middle and right column panels the barplots show snapshots for 2060 and 2099. The bar height corresponds to the average, and the gray vertical lines show the minimum-maximum range across all ensemble members, while the individual data points are also plotted. The multiplication and plus signs indicate expectations from scaling up CDR application and combining methods respectively, based on linearity assumptions (see Table 1). To aid visualization, the vertical axis is flipped in the top panel.*

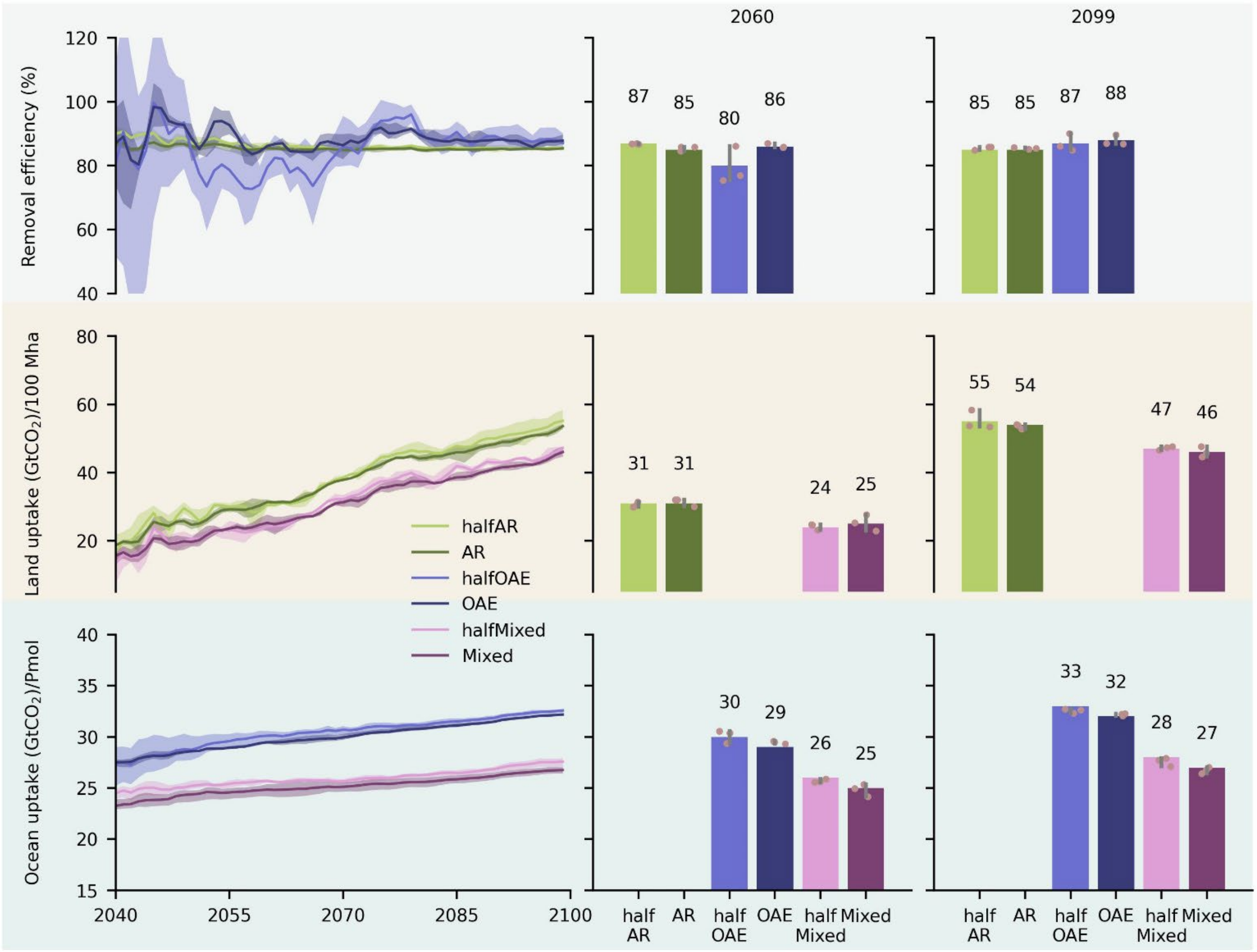

Figure S 3: ***Removal efficiency and uptake per unit of Carbon Dioxide Removal (CDR) application in MPI-ESM****: The left column panels show the timeseries of MPI-ESM average: (top) removal efficiency (see Methods, Table 1), (middle) land carbon uptake (GtCO2) per 100 Mha of planted forest, and (bottom) ocean carbon uptake (GtCO2) per Pmol of alkalinity added to the ocean surface. In all cases, uptake and removal refer to changes in carbon stocks compared to REF. The shading around the mean shows the minimum-maximum range across all ensemble members. To aid interpretation, in the middle and right column panels the barplots show snapshots for 2060 and 2099. The bar height corresponds to the model average, and the gray vertical lines show the minimum-maximum range across all ensemble members, while the individual data points are also plotted.*

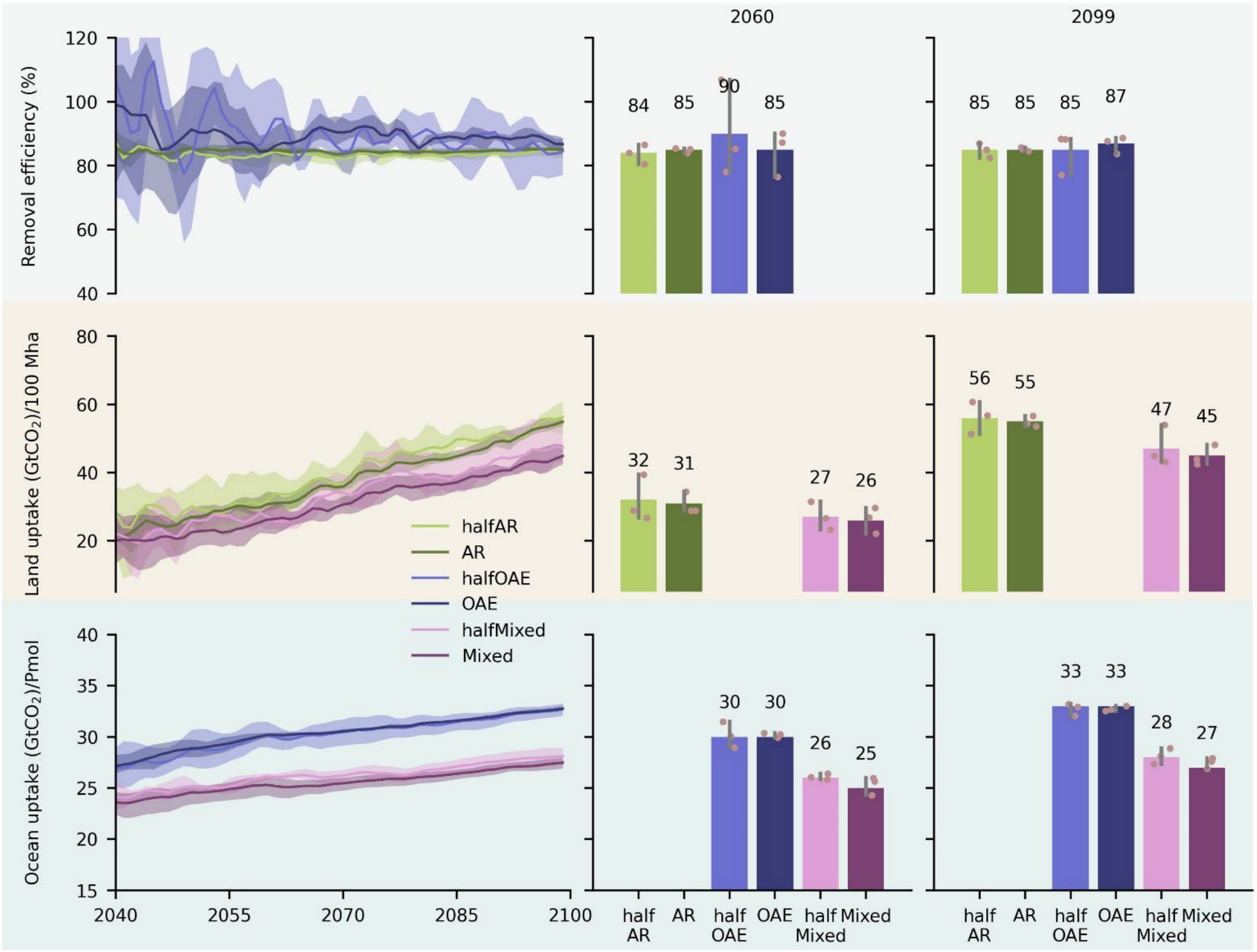

Figure S 4: ***Removal efficiency and uptake per unit of Carbon Dioxide Removal (CDR) application in FOCI****: The left column panels show the timeseries of FOCI average: (top) removal efficiency (see Methods, Table 1), (middle) land carbon uptake (GtCO2) per 100 Mha of planted forest, and (bottom) ocean carbon uptake (GtCO2) per Pmol of alkalinity added to the ocean surface. In all cases, uptake and removal refer to changes in carbon stocks compared to REF. The shading around the mean shows the minimum-maximum range across all ensemble members. To aid interpretation, in the middle and right column panels the barplots show snapshots for 2060 and 2099. The bar height corresponds to the model average, and the gray vertical lines show the minimum-maximum range across all ensemble members, while the individual data points are also plotted.*

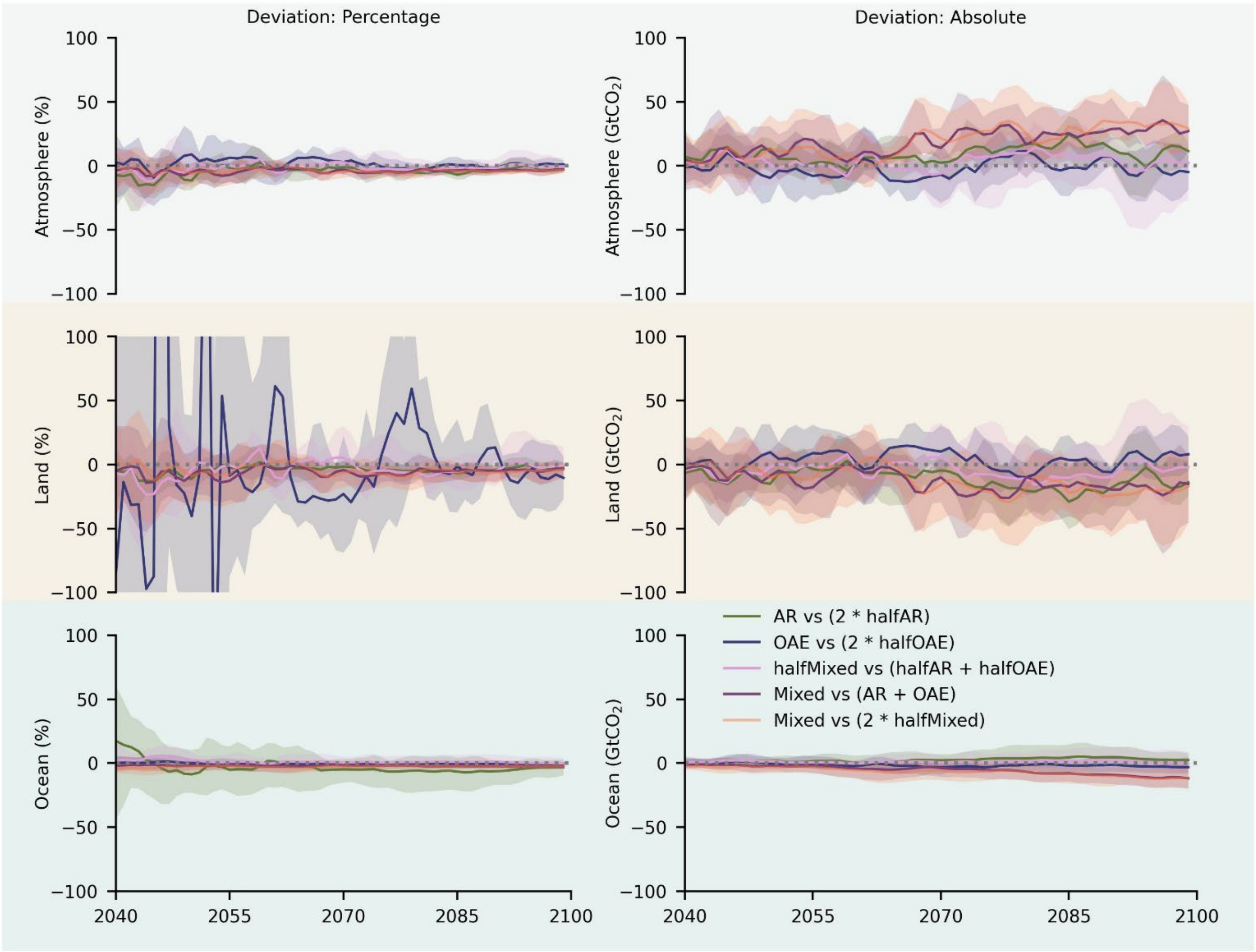

*Figure S 5: **Deviation from linearity**: The plots show the timeseries of intermodel average deviation from expectations based on linearity arguments (see Table 1) when scaling up and/or combining Carbon Dioxide Removal (CDR) methods in (top) atmosphere, (middle) land, and (bottom) ocean carbon responses. Deviation is expressed both (left) as a percentage (100*(realized flux – expectation)/expectation (%)), and (right) in absolute carbon amount (realized flux – expectation ($GtCO_2$)). The shading around the mean shows the minimum-maximum range across both models and all ensemble members.*

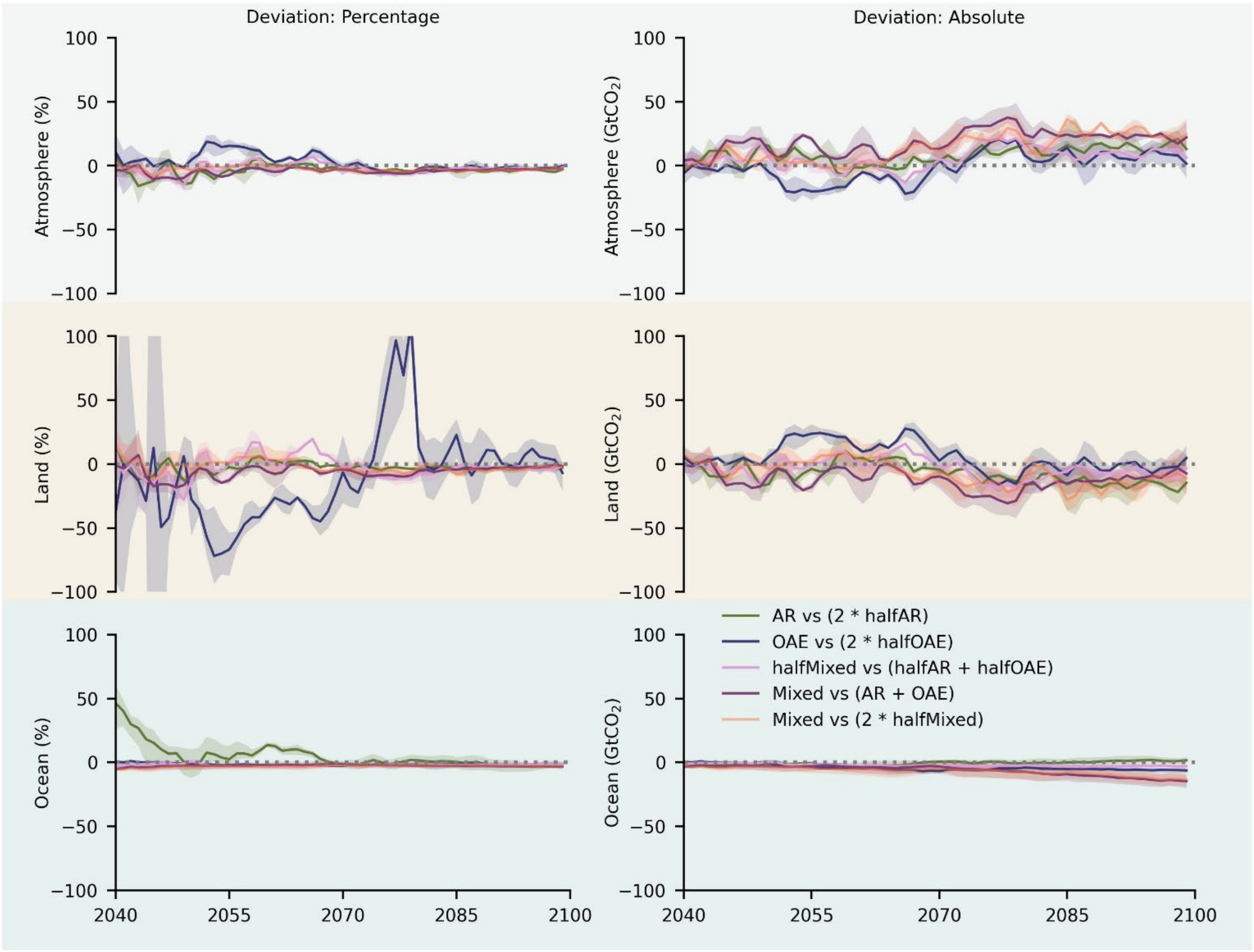

Figure S 6: ***Deviation from linearity in MPI-ESM****: The plots show the timeseries of MPI-ESM average deviation from expectations based on linearity arguments (see Table 1) when scaling up and/or combining Carbon Dioxide Removal (CDR) methods in (top) atmosphere, (middle) land, and (bottom) ocean carbon responses. Deviation is expressed both (left) as a percentage (100*(realized flux – expectation)/expectation (%)), and (right) in absolute C amount (realized flux – expectation (GtCO2)). The shading around the mean shows the minimum-maximum range across all ensemble members.*

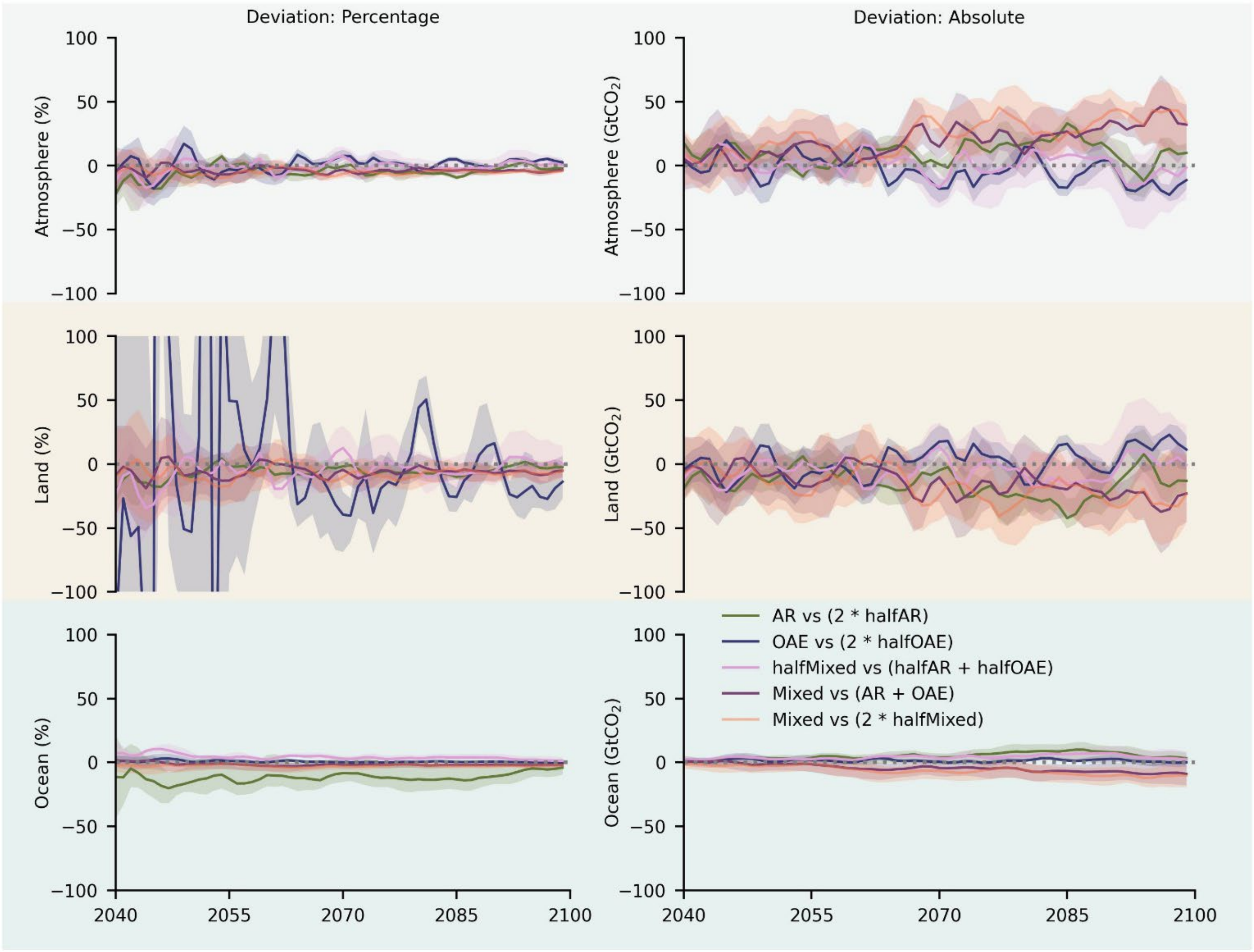

*Figure S 7: : **Deviation from linearity in FOCI**: The plots show the timeseries of FOCI average deviation from expectations based on linearity arguments (see Table 1) when scaling up and/or combining Carbon Dioxide Removal (CDR) methods in (top) atmosphere, (middle) land, and (bottom) ocean carbon responses. Deviation is expressed both (left) as a percentage (100*(realized flux – expectation)/expectation (%)), and (right) in absolute C amount (realized flux – expectation (GtCO2)). The shading around the mean shows the minimum-maximum range across all ensemble members.*

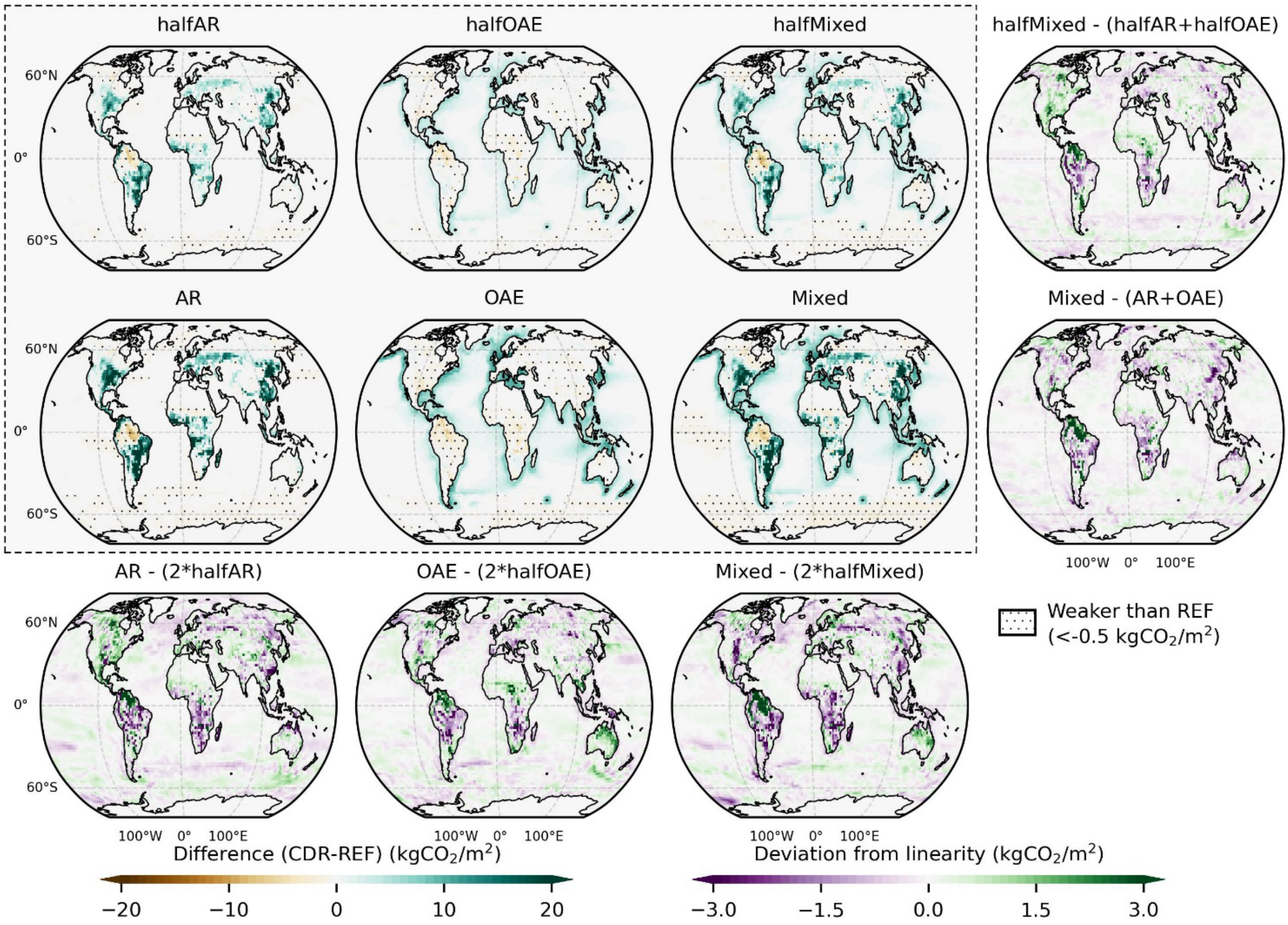

Figure S 8: ***Carbon fluxes at the gridcell level in MPI-ESM****: The maps within the shaded box show the MPI-ESM average difference in cumulative carbon flux (kgCO2/m2) between each Carbon Dioxide Removal (CDR) and the REF scenario by 2099. A positive value (shown in blue) indicates carbon sequestration on land and ocean compared to REF, while a negative value (shown in brown) suggests that the land or ocean carbon sink is reduced compared to REF. To aid interpretation, the hatching highlights the regions with a negative value less than -0.5 kgCO$_2$/m^2. The maps outside the shaded box show the deviation from linear expectations (see Table 1), as noted in the titles. A different scale and colormap are used, with positive values (shown in green) indicating that the realized flux (sequestration or weakening compared to REF) is higher than the linear expectation (sequestration or weakening compared to REF) (see Table 1). For example, in gridcells where both the realized and expected flux are negative (weakening compared to REF), a positive value suggests that the realized weakening is less strong in magnitude as the expected one.*

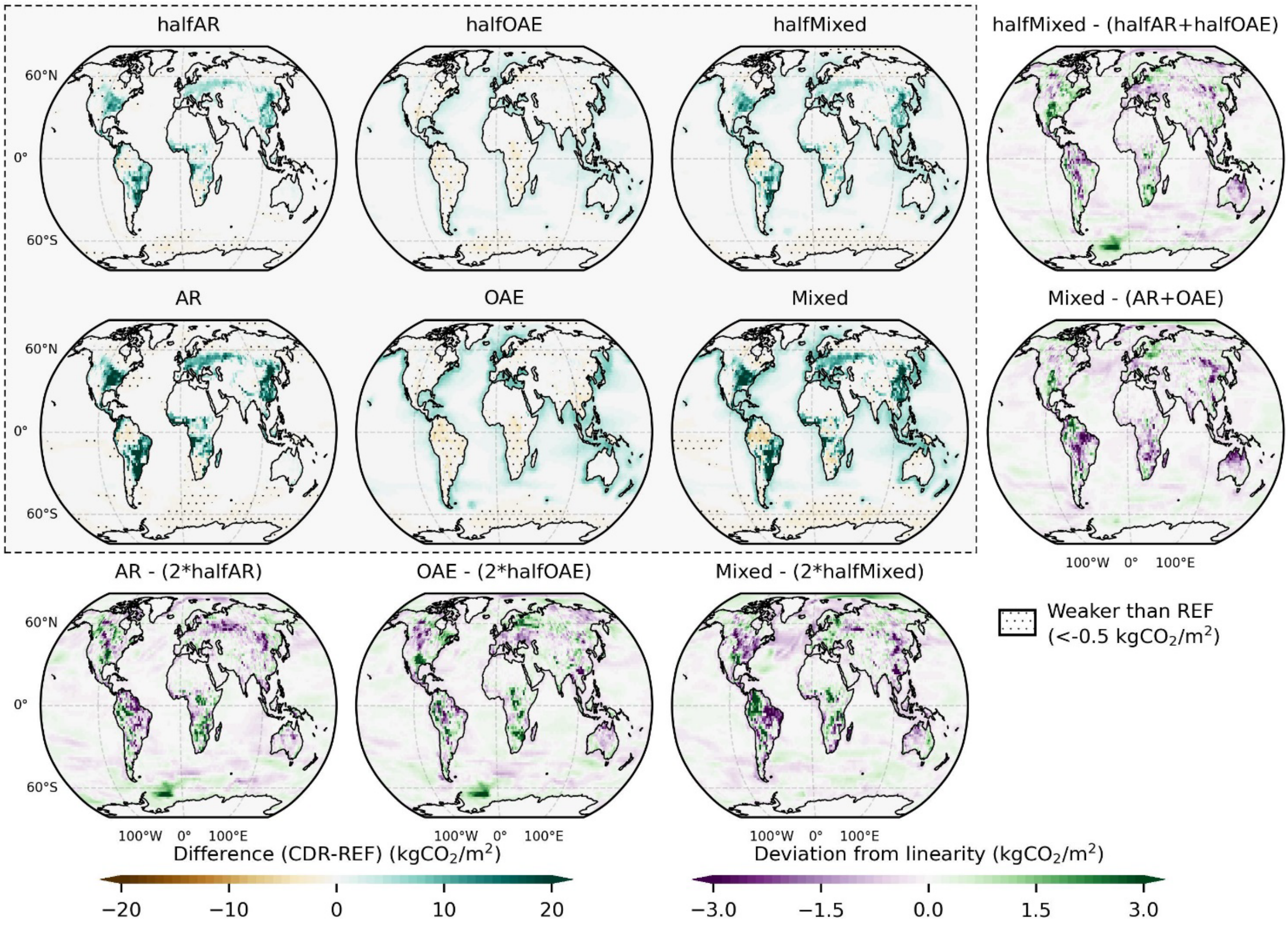

Figure S 9: ***Carbon fluxes at the gridcell level in FOCI****: The maps within the shaded box show the FOCI average difference in cumulative carbon flux (kgCO2/m2) between each Carbon Dioxide Removal (CDR) and the REF scenario by 2099. A positive value (shown in blue) indicates carbon sequestration on land and ocean compared to REF, while a negative value (shown in brown) suggests that the land or ocean carbon sink is reduced compared to REF. To aid interpretation, the hatching highlights the regions with a negative value less than -0.5 kgCO_2/m^2. The maps outside the shaded box show the deviation from linear expectations (see Table 1), as noted in the titles. A different scale and colormap are used, with positive values (shown in green) indicating that the realized flux (sequestration or weakening compared to REF) is higher than the linear expectation (sequestration or weakening compared to REF) (see Table 1). For example, in gridcells where both the realized and expected flux are negative (weakening compared to REF), a positive value suggests that the realized weakening is less strong in magnitude as the expected one.*

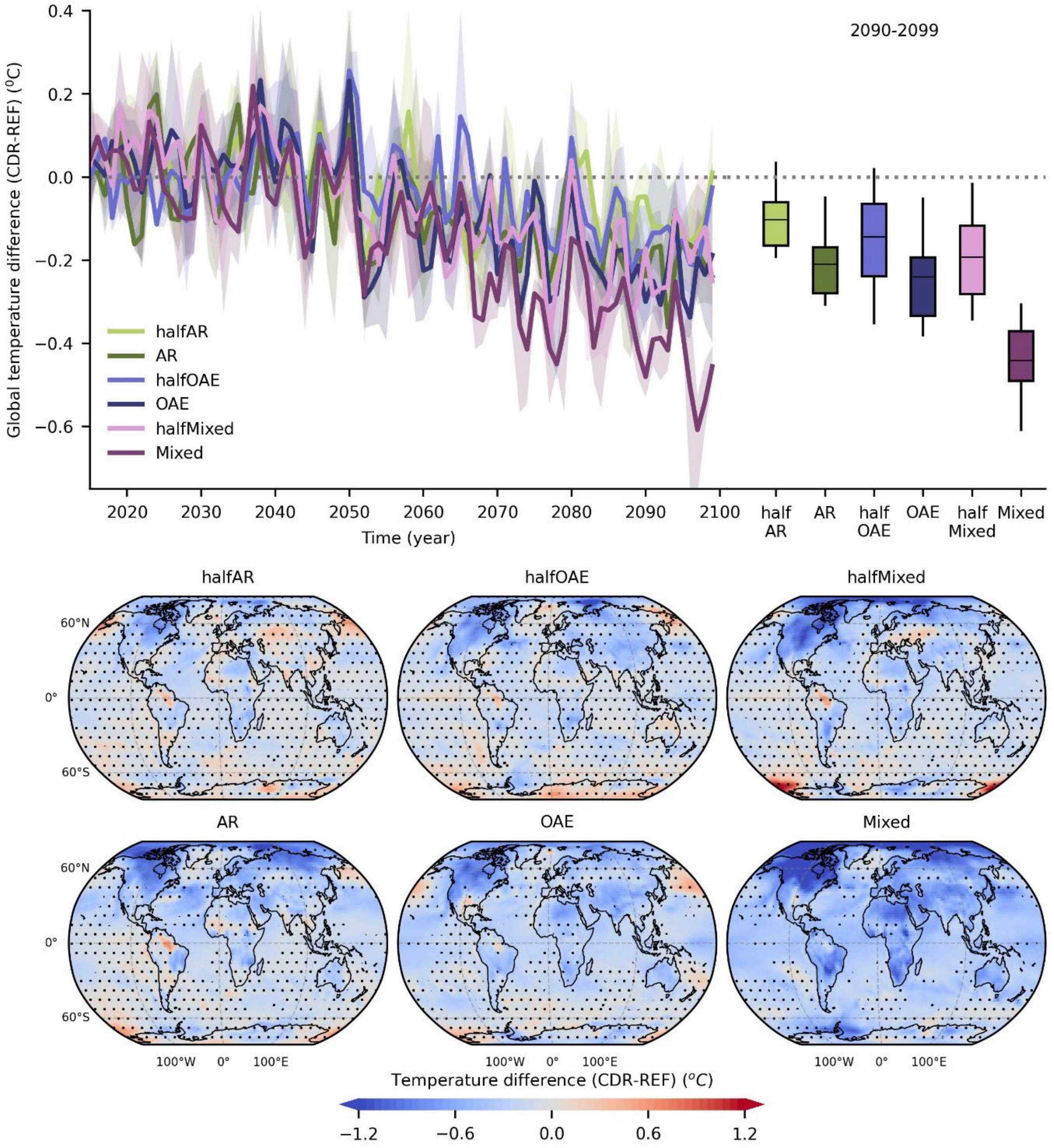

*Figure S 10: **Temperature mitigation in MPI-ESM**: (Top) For every Carbon Dioxide Removal (CDR) scenario the lineplots show the timeseries of the average difference in globally averaged 2m temperature (°C) compared to the mean temperature under REF in MPI-ESM. The shading around the mean shows the minimum-maximum range across all ensemble members. To aid interpretation, the boxplots on the right show yearly values of globally averaged temperature between 2090-2099 pooled from all ensemble members. The whiskers show the 5th-95th percentiles of pooled values. The average 2090-2099 temperature is shown with the horizontal black lines. (Bottom) The maps show the difference in average 2090-2099 2m temperature (°C) between each CDR and the REF scenario in MPI-ESM. Stippling highlights statistical insignificant differences. Statistical significance at the 10% significance level is declared based on a two-tailed Student's t-test adjusted to account for temporal lag-1 autocorrelation[12]. For each gridcell, the test is applied over the yearly data pooled together from all ensemble members for the given period.*

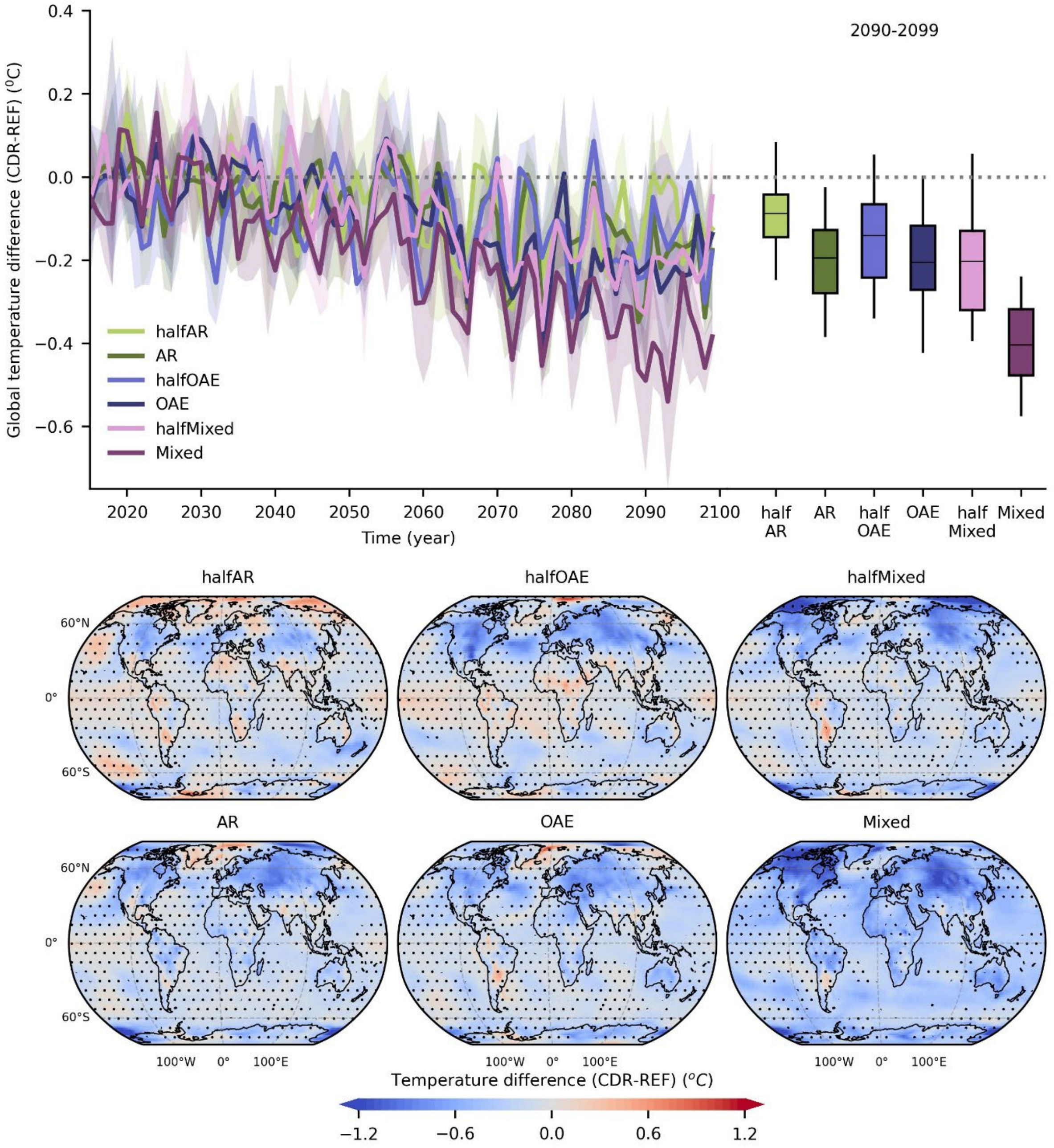

*Figure S 11: : **Temperature mitigation in FOCI**: (Top) For every Carbon Dioxide Removal (CDR) scenario the lineplots show the timeseries of the average difference in globally averaged 2m temperature (°C) compared to the mean temperature under REF in FOCI. The shading around the mean shows the minimum-maximum range across all ensemble members. To aid interpretation, the boxplots on the right show yearly values of globally averaged temperature between 2090-2099 pooled from all ensemble members. The whiskers show the 5th-95th percentiles of pooled values. The average 2090-2099 temperature is shown with the horizontal black lines. (Bottom) The maps show the difference in average 2090-2099 2m temperature (°C) between each CDR and the REF scenario in FOCI. Stippling highlights statistical insignificant differences. Statistical significance at the 10% significance level is declared based on a two-tailed Student's t-test adjusted to account for temporal lag-1 autocorrelation[12]. For each gridcell, the test is applied over the yearly data pooled together from all ensemble members for the given period.*

Supplementary References

1. Keller, D. P., Feng, E. Y. & Oschlies, A. Potential climate engineering effectiveness and side effects during a high carbon dioxide-emission scenario. *Nat Commun* **5**, 3304 (2014).
2. Feng, E. Y., Koeve, W., Keller, D. P. & Oschlies, A. Model-Based Assessment of the CO2 Sequestration Potential of Coastal Ocean Alkalinization. *Earth's Future* **5**, 1252–1266 (2017).
3. Lenton, A., Matear, R. J., Keller, D. P., Scott, V. & Vaughan, N. E. Assessing carbon dioxide removal through global and regional ocean alkalinization under high and low emission pathways. *Earth System Dynamics* **9**, 339–357 (2018).
4. Sonntag, S., Pongratz, J., Reick, C. H. & Schmidt, H. Reforestation in a high-CO2 world—Higher mitigation potential than expected, lower adaptation potential than hoped for. *Geophysical Research Letters* **43**, 6546–6553 (2016).
5. Sonntag, S. *et al.* Quantifying and Comparing Effects of Climate Engineering Methods on the Earth System. *Earth's Future* **6**, 149–168 (2018).
6. Koch, A., Brierley, C. & Lewis, S. L. Effects of Earth system feedbacks on the potential mitigation of large-scale tropical forest restoration. *Biogeosciences* **18**, 2627–2647 (2021).
7. Loughran, T. F. *et al.* Limited Mitigation Potential of Forestation Under a High Emissions Scenario: Results From Multi-Model and Single Model Ensembles. *Journal of Geophysical Research: Biogeosciences* **128**, e2023JG007605 (2023).
8. Palmiéri, J. & Yool, A. Global-Scale Evaluation of Coastal Ocean Alkalinity Enhancement in a Fully Coupled Earth System Model. *Earth's Future* **12**, e2023EF004018 (2024).
9. Moustakis, Y., Nützel, T., Wey, H.-W., Bao, W. & Pongratz, J. Temperature overshoot responses to ambitious forestation in an Earth System Model. *Nat Commun* **15**, 8235 (2024).
10. Jeltsch-Thömmes, A. *et al.* Earth system responses to carbon dioxide removal as exemplified by ocean alkalinity enhancement: tradeoffs and lags. *Environ. Res. Lett.* **19**, 054054 (2024).
11. Wey, H.-W. *et al.* CMIP6 Models agree on similar carbon cycle feedbacks between enhancing terrestrial and marine carbon sinks. *Environ. Res. Lett.* **20**, 054029 (2025).
12. Zwiers, F. W. & von Storch, H. Taking Serial Correlation into Account in Tests of the Mean. *Journal of Climate* **8**, 336–351 (1995).
